# Urethral Caruncle and Urethral Prolapse

**Published:** 1908-06-27

**Authors:** 


### Gynæcology.

### URETHRAL CARUNCLE AND URETHRAL PROLAPSE.

Although the diagnosis of urethral caruncle presents no difficulties as a rule, there are conditions which are from time to time mistaken for it, and errors of treatment in this way arise. Prolapse of the urethral mucous membrane is an uncommon affection, but if carefully examined should not be overlooked or mistaken for any other condition. Here can be seen a complete or partial ring of protruding mucous membrane, with a central instead of an excentric dimple marking the orifice of the urethra. The actual amount of protrusion is variable but may be quite considerable, and then there is some risk of strangulation occurring, with sloughing of the exposed part. In any case the dimpled arrangement and the passage of a sound into it will serve to distinguish prolapse from a true caruncle. The latter, it must not be forgotten, is a discrete tumour growing always from the posterior wall of the urethra and having usually a somewhat wide base. The colour is bright crimson as compared with the dull pink of the rest of the mucous membrane. The size of a caruncle may reach half to three-quarters of an inch in length, but they are nearly always laterally compressed if of any size, on account of the apposition of the labia minora. Both caruncles and prolapse produce a certain amount of discharge and are liable to bleed if touched; hence bleeding on coitus or on straining are common symptoms of both. Pain is associated with both, but it must not be forgotten that some caruncles are painless, others exquisitely painful to the touch. The cause of this pain is not clear, for no nerve fibres can be demonstrated in caruncles (H. Williamson), but it is probably connected with the disturbance and inflammatory change around the nerve endings at the base of the growth. The same explanation accounts for the pain associated with urethral prolapse. The only other condition which at all could be conceived to be like a caruncle or a prolapse is the vascular condition surrounding the urethral orifice in elderly women. In this condition there is the same pain with some discharge, and the scalding or pricking pain on micturition. In simple vascularity the orifice is usually gaping, with exposure of the mucous membrane, but there is no actual protrusion and nothing which could be called a new growth. This vascularity is very common, and appears to be the result of atrophic changes associated with simple catarrhal vulvitis. The lesion is due to superficial desquamation around the meatus urinarius, with consequent exposure of the underlying capillary loops. As these are no longer supported and compressed from without they become dilated and assume a bright vascularity.

The causation of urethral caruncle is not always clear, but it must be primarily the result of inflammatory changes in some small traumatism, such as a scratch. They have been ascribed to gonorrhœa by some observers, and although it is no doubt true that this disease is responsible for them in many instances, it cannot always be so. As the mass is always of the nature of a granuloma, it is clear that other organisms besides the gonococcus may be responsible for it. Histologically, the tumour consists of granulation tissue with widely dilated vessels, and according to the amount of fibrous tissue produced, so we occasionally find caruncles more fibrous and less angiomatous than usual. They are always more or less covered by stratified epithelium, and in some instances this produces gland-like in-dippings of the surface, producing the so-called glandular caruncles. The pathology of urethral prolapse is even more obscure. It may occur in quite young children or in women at all ages. It is usually associated with straining in defæcation or in performing heavy work, but it is by no means clear why the urethral mucous membrane should prolapse from these causes. We should expect the anal or vaginal prolapses to occur before the urethral. As a matter of clinical experience the urethral prolapse may occur alone.

The treatment of a urethral caruncle is often disappointing, for recurrence is very common. It is especially common in cases where the growth has been removed with the cautery. The reason for this is not far to seek; the cautery leaves behind a surface covered by an eschar which soon separates leaving a granulating surface, and from this a new granuloma is quickly formed. A caruncle in this way may recur in four to six weeks after removal. The best way to deal with these growths is to excise them by cutting out a small wedge of tissue at the base bringing the edges together with a couple of fine sutures. Care must be taken that the sutures include any bleeding point, otherwise a small hæmatoma may form. In one case known to the writer, gangrene of the tissues over a small hæmatoma thus formed occurred, and gave rise to a prolonged period of healing. Excision, too, is the best treatment for prolapse of the mucous membrane, and is a very troublesome little operation. There is free capillary bleeding when the protruding portion is cut off, and it is exceedingly difficult to introduce the sutures so as to bring the mucous membrane of the urethra into exact apposition with that of the vulva. The final result, however, after accurate suture is very good, and the subsequent cicatrisation seems to prevent a recurrence of the condition. In one case in a small child, superficial sloughing of the prolapsed parts occurred from strangulation. After several weeks of careful dressing with iodoform powder, the small ulcer healed and the cicatrisation cured the condition. For the simple vascular condition around the meatus, palliative treatment generally suffices, the desired result being to bring about the formation of fresh epithelial layers. This can be done by the application of stimulating antiseptic lotions, the best being the lotio nigra B.P. This can be kept applied upon lint, frequently changed and kept in place by a small pad inside a sanitary towel or diaper.

